# hTERT-immortalized mouse alveolar macrophages retain primary-like transcriptional and functional programs

**DOI:** 10.1007/s44307-026-00130-x

**Published:** 2026-09-23

**Authors:** Xinyi Hu, Youxiang Cheng, Zekai Qian, Zhen Li, Yixiao Liang, Huan Wu, Wenwen Song, Anlong Xu, Yong Ge, Shaochun Yuan

**Affiliations:** 1 Guangdong Province Key Laboratory of Pharmaceutical Functional Genes, MOE Key Laboratory of Gene Function and Regulation, MOE Engineering Center of South China Sea Marine Biotechnology, State Key Laboratory of Biocontrol, School of Life Sciences, Sun Yat-Sen University, Guangzhou, 510275 P.R. China; 2 Laboratory for Marine Biology and Biotechnology, Pilot National Laboratory for Marine Science and Technology, Qingdao, 266237 P.R. China; 3 Sun Yat-sen University Institute of Advanced Studies Hong Kong, Hong Kong, 999077 P.R. China

## Introduction

Alveolar macrophages (AMs) are the most abundant immune cells in the pulmonary alveoli, serving as the first line of defense against respiratory pathogens while maintaining lung homeostasis (Ginhoux and Guilliams 2016; Hussell and Bell 2014; Varol et al. 2015). Under steady-state conditions, AMs exhibit an immunosuppressive phenotype that prevents unwarranted inflammation to harmless stimuli; upon encountering pathogens, they mount inflammatory responses including antigen presentation, neutrophil recruitment, and pro-inflammatory cytokine production (Allard et al. 2018). Beyond these roles, AMs contribute to the pathogenesis of various pulmonary diseases, underscoring the importance of understanding their biology (Aegerter et al. 2022). However, progress has been constrained by the limited availability of primary AMs, as their isolation requires bronchial lavage of experimental animals and yields only 200,000–500,000 cells per mouse, which restricts downstream applications (Busch et al. 2019).

To address this need, several AM cell lines have been developed. The AMJ2-C8 and AMJ2-C11 lines, generated by v-*raf*/v-*myc* oncogene transduction, exhibit aberrant responses to LPS and MDP and fail to secrete sufficient IL-1 and TNF (Palleroni et al. 1991). The widely used MH-S line, immortalized with SV40 T antigen, lacks the AM marker Siglec-F and fails to express CD116, rendering it unresponsive to GM-CSF and hyporesponsive to LPS (Brenner et al. 2016; Sankaran and Herscowitz 1995). Alternatively, AM-like cells can be differentiated from bone marrow precursors using GM-CSF, TGF-β, and rosiglitazone (Luo et al. 2021). However, these cells are not immortalized and have limited expansion capacity. Thus, existing models have failed to fully recapitulate primary AM characteristics.

Telomerase reverse transcriptase (hTERT) has been successfully used to immortalize various primary cell types without altering their phenotype (Jiang et al. 1999). Here, we generated an hTERT-immortalized AM (iAM) cell line derived from primary mouse AMs. We evaluated its proliferation, surface marker expression, transcriptional profile, and functional activities including phagocytosis, chemotaxis, and cytokine production. Furthermore, we assessed in vivo functionality by adoptive transfer into AM-depleted mice. Our results demonstrate that iAM cells proliferate stably, retain key phenotypic and transcriptional features of primary AMs, partially recapitulate hallmark AM functions, and restore pulmonary defense in vivo, providing a useful cellular system for studying AM biology.

## Materials and methods

C57BL/6 mice (6–8 weeks old) were purchased from the Sun Yat-sen University Laboratory Animal Center, and animal experiments were approved by the Institutional Animal Care and Use Committee (IACUC) of Sun Yat-sen University. All mice were maintained under specific-pathogen-free (SPF) conditions with a 12-h light/dark cycle and free access to food and water. Primary AMs were isolated from bronchoalveolar lavage fluid (BALF) as described (Luo et al. 2021) and cultured in RPMI-1640 with 10% FBS, 1% penicillin–streptomycin, 1 mM sodium pyruvate, and 20 ng/mL GM-CSF. iAM cells were generated by transducing primary AMs with hTERT-encoding lentivirus (MOI: 10), followed by puromycin selection and monoclonal isolation. Any other materials and methods described in this study are available in the Supplementary Information.

## Results

**Fig. 1:**
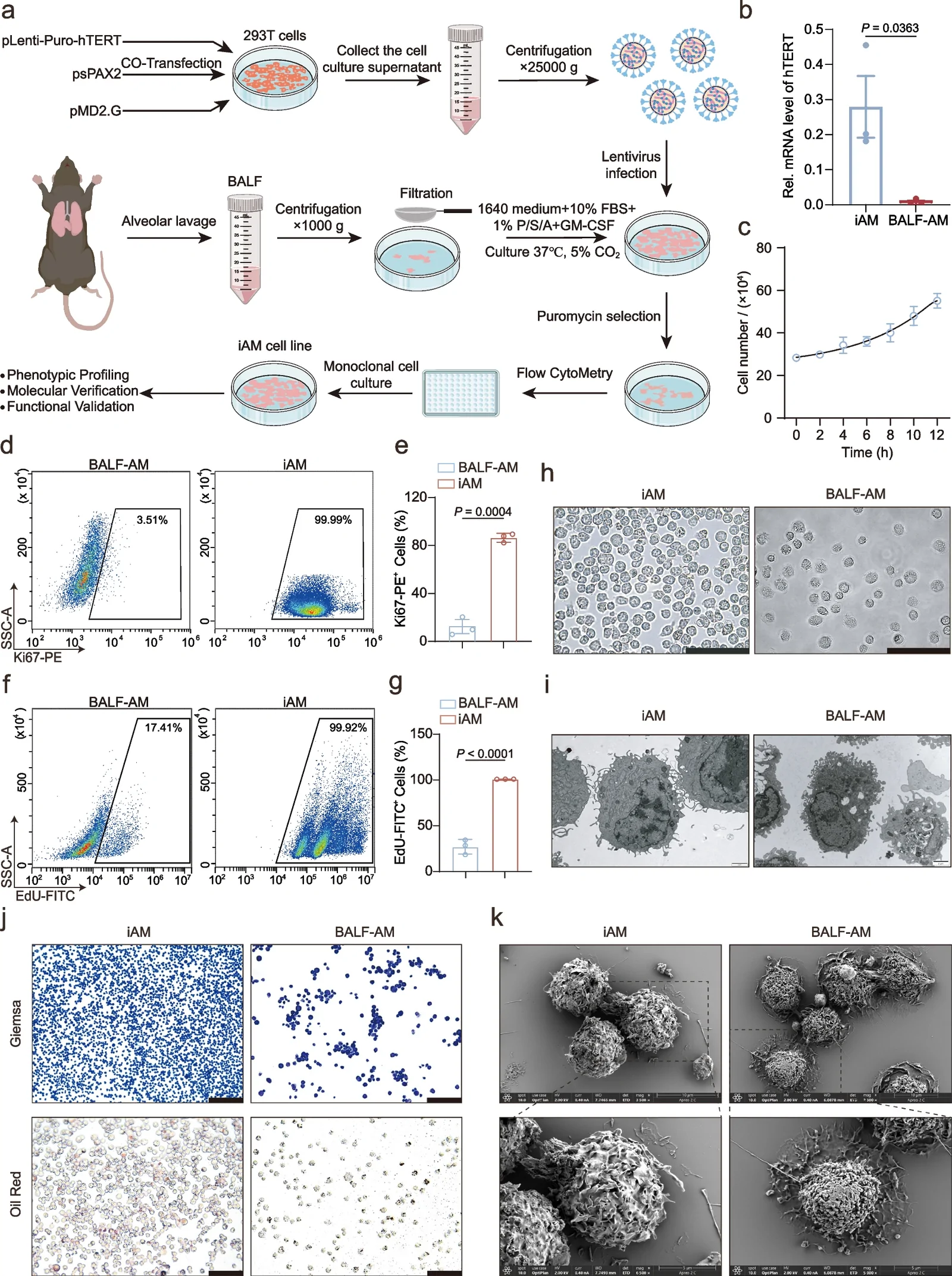
Establishment of iAM cells by hTERT overexpression and characterization of their proliferation and morphology. **a** Schematic diagram of the experimental workflow for immortalizing primary alveolar macrophages (AMs) using hTERT. **b** RNA-seq detecting hTERT expression in immortalized and primary AM cells. **c** iAM cell numbers were determined at the indicated time points during in vitro expansion. **d-e** Flow cytometry showing the expression of Ki67 protein in primary AM cells and iAM cell lines (**d**) and quantification of the percentage of Ki67^+^ cells (**e**). **f-g** Flow cytometry showing EdU incorporation in primary AM cells and iAM cell lines (**f**) and quantification of the percentage of EdU^+^ cells (**g**). **h** Bright-field images of primary AM cells and iAM cell lines. Scale bars, 50 μm. **i** Transmission electron microscope (TEM) micrographs of primary AM cells and iAM cell lines. Scale bars, 2 μm. **j** Bright field images of Giemsa (Upper panel) and Oil red (Lower panel) staining in primary AM cells and iAM cell lines. Scale bars, 100 μm. **k** Scanning electron microscope micrographs of primary AM cell and iAM cell lines during in vitro culture. The enlarged image shows an image of a single cell. Scale bars, 10 μm. Data are presented as the mean ± s.d. in **b**, **e**, **g** with individual measurements overlaid as dots, and statistical analysis was performed in **b**, **e**, **g** using a two-tailed Student’s *t*-test. All experiments were performed using iAM cells at passages 1–12 (P1–P12)

To overcome the limited proliferative capacity of primary alveolar macrophages (AMs), which undergo rapid senescence in culture, and the limitations of existing cell lines that lack key characteristics of primary cells, we immortalized primary mouse AMs via exogenous hTERT expression using a lentiviral system. After puromycin selection and monoclonal isolation, four stable hTERT-immortalized AM (iAM) cell lines were established (Fig. 1a). All four clones were confirmed to be mycoplasma‑free by PCR (Fig. S1) and exhibited comparable morphology, surface marker expression, and proliferative capacity. One representative clone was therefore selected for all subsequent experiments. STR profiling confirmed that the iAM cell line originated from the primary AMs (Table S1). hTERT mRNA was detectable exclusively in iAMs, but not in primary AMs (Fig. 1b). iAMs exhibited active proliferation with a doubling time of approximately 12 h (Fig. 1c), and nearly all iAMs were Ki67⁺ (Fig. 1d, e) and showed significantly higher EdU incorporation compared to primary cells (Fig. 1f, g), confirming their sustained proliferative capacity. Morphologically, iAMs resembled primary AMs under bright-field and transmission electron microscopy (Fig. 1h, i), with similar staining patterns for Giemsa and Oil Red O (Fig. 1j). Scanning electron microscopy revealed that iAMs maintained the spherical morphology of primary AMs, though with reduced pseudopod formation (Fig. 1k).

**Fig. 2:**
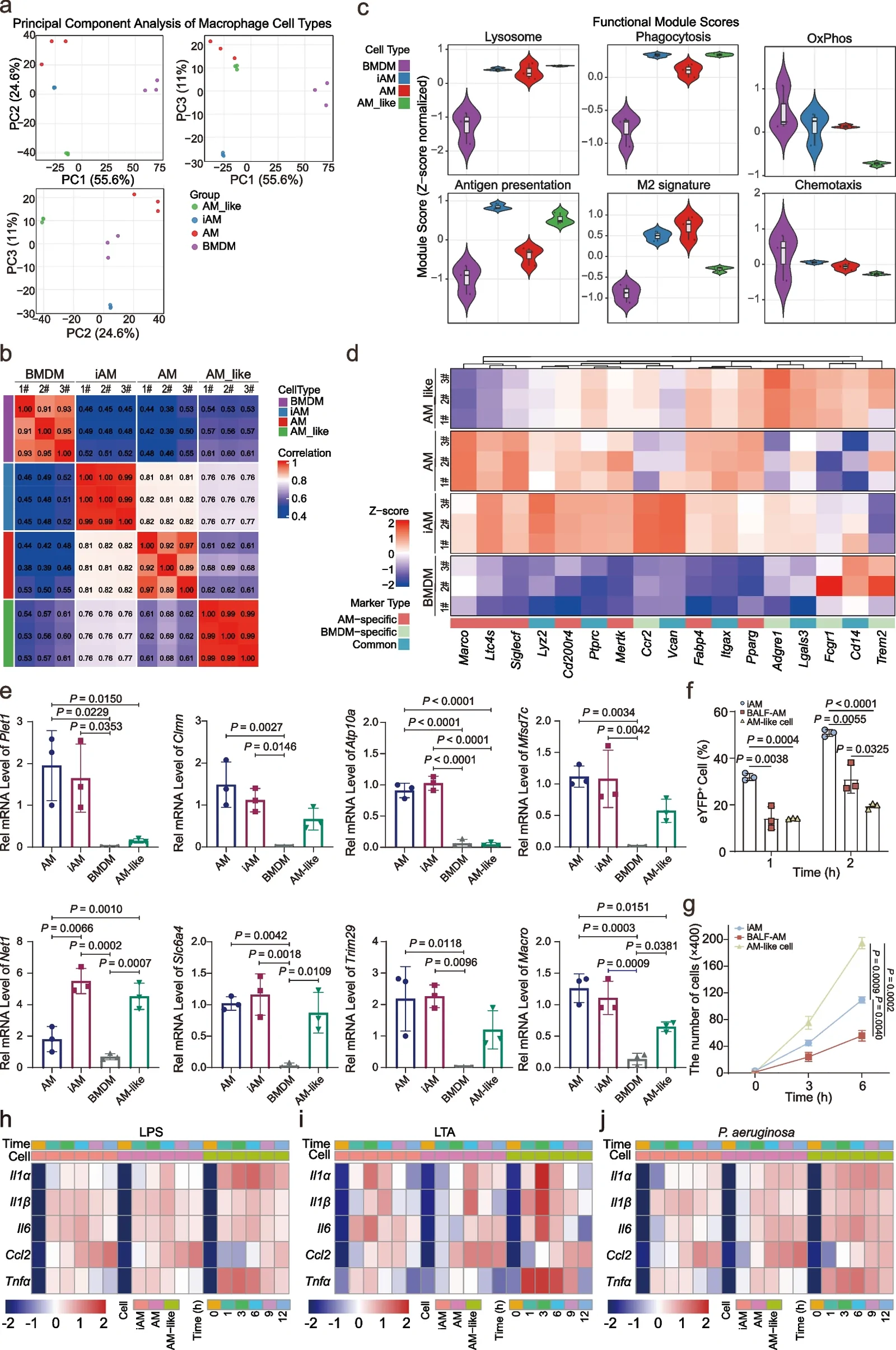
Transcriptional, molecular and functional validation of iAM cells fidelity to primary alveolar macrophages. **a** Principal component analysis (PCA) of bulk RNA-seq data from primary AM cells, iAM cells, AM-like cells, and BMDM cells. Three biological replicates per cell type. **b** Pairwise correlation heatmap (Pearson's correlation) showing global transcriptional similarities among the four cell types. **c** Functional module scoring showing the enrichment scores for lysosome, phagocytosis, oxidative phosphorylation (OxPhos), antigen presentation, M2 signature, and chemotaxis. **d** Targeted heatmap showing the expression of AM-specific, BMDM-specific, and pan-macrophage marker genes in primary AM cells, iAM cells, AM-like cells, and BMDM cells. **e** qRT-PCR showing the expression of AM-specific genes, alternative activation signature genes, and self-renewal signature genes in primary AM cells, iAM cells, BMDM cells, and AM-like cells, and quantification of relative mRNA levels. **f** Quantification of phagocytosis by flow cytometry, showing the percentage of eYFP⁺ iAM, primary AM, and AM-like cells after incubation with eYFP-labeled *E. coli*. **g** Transwell chemotaxis assay quantifying migrated iAM, primary AM, and AM-like cells in response to *P. aeruginosa*. **h-j** qRT-PCR showing the relative expression levels of *Il1α*, *Il1β*, *Il6*, *Ccl2*, and *Tnfα*, in iAM cells, primary AM cells, and AM-like cells after stimulation with LPS (**h**), LTA (**i**), and inactivated *P. aeruginosa* (**j**). Data are presented as the mean ± s.d. in **e**, **f** and **g** with individual measurements overlaid as dots. Statistical analysis was performed using multiple comparisons following one-way ANOVA in **e**, and multiple comparisons following two-way ANOVA in **f** and **g**. All experiments were performed using iAM cells at passages 1–12 (P1–P12)

We next performed bulk RNA-seq to compare iAMs, primary AMs, AM-like cells (Luo et al. 2021), and BMDMs. Principal component analysis (PCA) showed that iAMs clustered more closely to primary AMs than to AM-like cells or BMDMs (Fig. 2a), with a substantially smaller transcriptional distance between AM and iAM than between AM and BMDM or iAM and BMDM (Table S2). Pairwise correlation analysis confirmed higher transcriptional similarity of iAMs to primary AMs (Fig. 2b). Hierarchical clustering heatmap further revealed that iAMs displayed expression patterns highly reminiscent of primary AMs across the majority of gene modules (Fig. S2). Differential expression analysis identified a total of 2,953 DEGs (|log_2_FC| ≥ 1 and *P*.adj < 0.05) between iAMs and primary AMs, with 1,440 genes upregulated and 1,513 downregulated in iAMs (Fig. S3a). KEGG enrichment analysis revealed that the DEGs were mainly enriched in pathways related to immune signaling and metabolism (Fig. S3b). Functional module scoring revealed that iAMs closely mirrored primary AMs across lysosome, phagocytosis, oxidative phosphorylation, chemotaxis, and M2 signature, whereas antigen presentation scored higher in iAMs (Fig. 2c). Expression of genes associated with AM-relevant biological pathways also showed high similarity between iAMs and primary AMs (Fig. S3c). Targeted heatmap analysis demonstrated that iAMs robustly expressed AM-specific markers, whereas AM-like cells exhibited a mixed AM/BMDM signature (Fig. 2d). Flow cytometric analysis further confirmed that iAMs retained the characteristic AM surface phenotype, including F4/80^int^, CD11b^low^, Siglec-F^high^, and CD11c^high^ (Fig. S3d, e). Meanwhile, qRT-PCR validated that iAMs expressed comparable levels of AM-specific, alternative activation, and self-renewal genes to primary AMs (Fig. 2e). Phagocytosis assay using eYFP-labeled *E. coli* also revealed that iAMs exhibited superior phagocytic activity compared to primary AMs and AM-like cells (Fig. 2f; Fig. S4a). Transwell chemotaxis assays showed that iAMs displayed enhanced migratory capacity toward *P. aeruginosa* relative to primary AMs (Fig. 2g; Fig. S4b, c). Furthermore, iAMs responded to LPS, LTA, or inactivated *P. aeruginosa* with inflammatory responses comparable to those of primary AMs (Fig. 2h-j). Hence, iAMs exhibit typical phagocytosis, chemotaxis and inflammatory sensitivity, just like primary AMs.

**Fig. 3:**
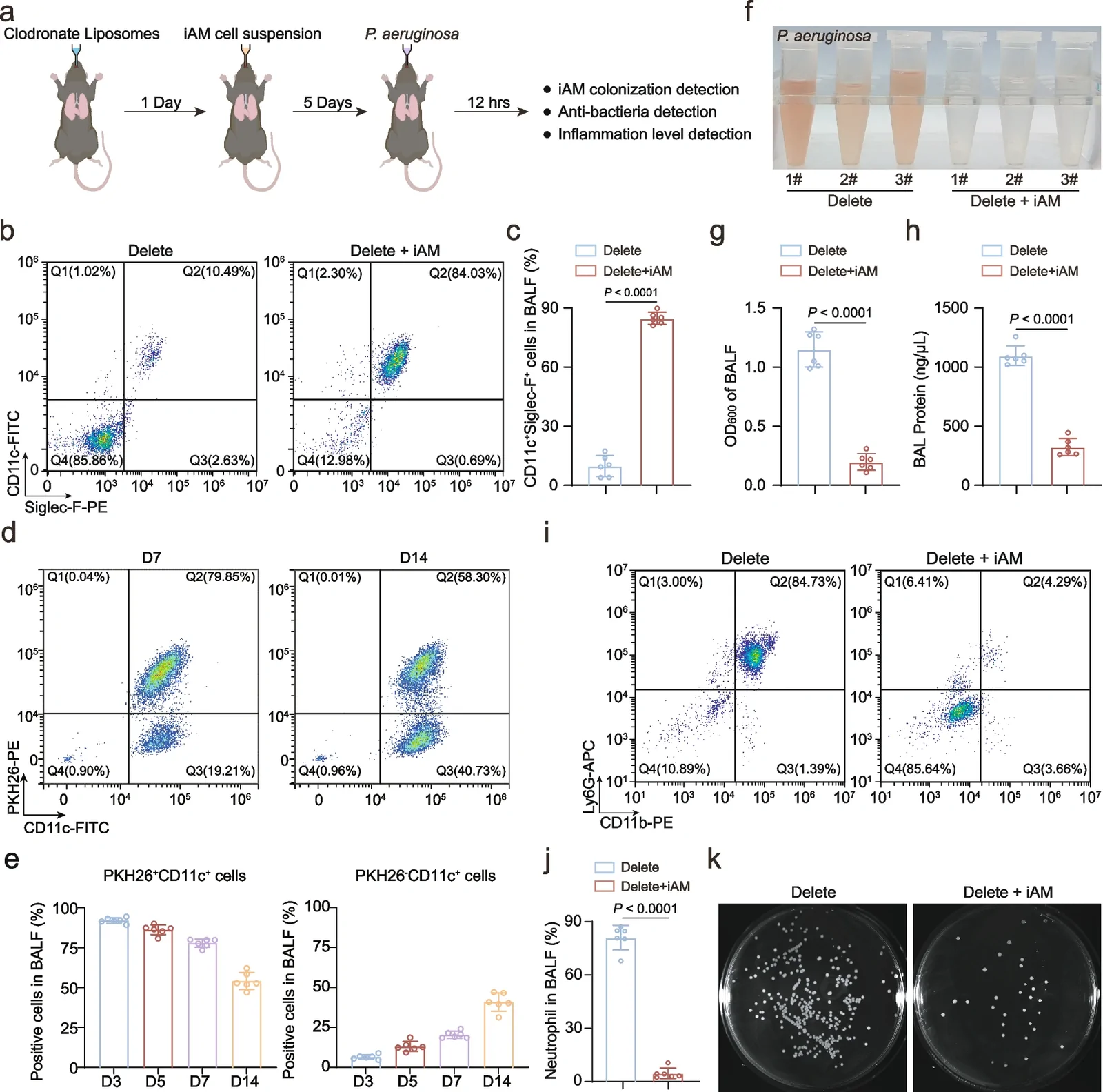
iAM cells reconstitute pulmonary antibacterial defense in vivo. **a** Experimental schema of alveolar macrophage depletion and reconstitution using clodronate liposomes followed by intratracheal challenge with 1.5 × 10^7^ CFU of *P. aeruginosa* per mouse. **b-c** Flow cytometry showing iAM cell engraftment in the lungs after intratracheal transfer. Representative plots of CD11c⁺ Siglec-F⁺ cells are shown in (**b**), and quantification of engraftment efficiency (percentage of CD11c⁺ Siglec-F⁺ cells) is shown in (**c**). *n* = 6 mice per group. **d-e** Flow cytometry showing iAM cell engraftment in the lungs after intratracheal transfer. Representative plots of PKH26⁺CD11c⁺ and PKH26⁻CD11c⁺ cells are shown in (**d**), and quantification of the percentage of PKH26⁺CD11c⁺ and PKH26⁻CD11c⁺ cells at each time point is shown in (**e**). *n* = 6 mice per time point. **f-g** Representative images of BALF appearance (**f**) and measurement of optical density at 600 nm (OD_600_) (**g**) from AM-depleted or iAM-reconstituted mice at 12 h after *P. aeruginosa* administration. *n* = 6 mice per group. **h** Quantification of BALF protein from AM-depleted or iAM-reconstituted mice at 12 h after *P. aeruginosa* administration. *n* = 6 mice per group. **i-j** Flow cytometry showing CD11b⁺ Ly6G⁺ neutrophil recruitment in BALFs from AM-depleted or iAM-reconstituted mice at 12 h after *P. aeruginosa* administration. Representative plots are shown in (**i**), and quantification of the percentage of neutrophils is shown in (**j**). *n* = 6 mice per group. **k** Representative bacterial plate images of BALFs from AM-depleted or iAM-reconstituted mice at 12 h after *P. aeruginosa* administration. Data pooled from two independent experiments are presented as mean ± SD in **c**, **e**, **g**, **h**, and **j**. Statistical analysis was performed using a two-tailed Student’s *t*-test. All experiments were performed using iAM cells at passages 1–12 (P1–P12)

To evaluate the physiological relevance of iAMs, we established an AM depletion and reconstitution model using clodronate liposomes followed by *P. aeruginosa* challenge (Fig. 3a). Flow cytometric analysis showed that resident AMs (CD11c^+^Siglec-F^+^ cells) constituted 94.7% ± 0.9% of total BALF cells before clodronate liposome treatment and decreased to 4.1% ± 1.4% after depletion, corresponding to an approximately 95.7% depletion efficiency (Fig. S5a, b). Intratracheal transfer of iAMs resulted in stable engraftment in the alveolar space (Fig. 3b, c). To evaluate the long-term persistence of engrafted iAMs, we labeled iAMs with PKH26 prior to transfer and tracked them by flow cytometry at multiple time points post-transfer. PKH26^+^ iAMs were detected throughout the 14-day observation period (Fig. 3d, e; Fig. S5c), with transferred PKH26^+^ iAMs accounting for more than 50% of the CD11c^+^ population at day 14 (Fig. 3e). AM-depleted mice exhibited severe lung injury, as evidenced by blood-tinged BALF, increased BALF turbidity (OD₆₀₀), elevated total protein levels, excessive neutrophil infiltration, and high bacterial burden (Fig. 3f-k). iAM reconstitution significantly alleviated all these pathological features, restoring bacterial clearance and limiting neutrophil recruitment. These results indicate that iAM cells can functionally replace endogenous AMs in pulmonary host defense.

## Discussion

Although BMDMs and monocyte-derived macrophages (MDMs) have long served as surrogates, these models fundamentally differ from tissue-resident macrophages in their ontogeny, transcriptional programming, and functional repertoire (Garbi and Lambrecht 2017; Ginhoux and Guilliams 2016; Varol et al. 2015). Thus, one of the most persistent challenges in macrophage biology is the lack of cell models that accurately reflect the tissue-specific identity of resident macrophages. This bottleneck is compounded by the difficulty in obtaining primary AMs and the absence of robust in vitro alternatives, since primary AMs rapidly lose their phenotypic characteristics upon ex vivo culture (Subramanian et al. 2022). Existing AM cell lines such as MH-S, AMJ2-C8, and AMJ2-C11 have undergone oncogene-mediated immortalization that alters their transcriptional programming and leads to aberrant functional responses, including loss of canonical AM surface markers and abnormal cytokine production (Brenner et al. 2016; Palleroni et al. 1991; Sankaran and Herscowitz 1995). Here, we generated an hTERT-immortalized AM (iAM) cell line that maintains stable proliferation while retaining key AM surface markers (Siglec-F⁺ CD11c⁺ F4/80⁺ CD11bˡᵒʷ) and expressing AM-specific genes. Functionally, iAM cells recapitulate hallmark activities of primary AMs, including phagocytosis, chemotaxis, and cytokine production. Moreover, adoptive transfer into AM-depleted mice resulted in successful engraftment and ameliorated *P. aeruginosa*-induced lung injury, addressing key limitations of oncogene-immortalized lines.

Several limitations of the iAM model warrant consideration. AMs serve as central regulators of lung inflammation and are subject to intricate feedback mechanisms that modulate their functional states (Lambrecht 2006). Although iAMs recapitulate many phenotypic features of primary AMs, they do not fully mirror all functional properties of freshly isolated cells; for instance, they exhibit enhanced antigen-presenting capacity and fewer pseudopodia compared with primary AMs. In addition, immortalization and prolonged in vitro expansion may introduce genetic or epigenetic alterations and promote clonal selection, potentially affecting cellular phenotypes over extended culture periods (Subramanian et al. 2022). Although our data support the phenotypic and functional stability of iAMs over the passages examined, their long-term genetic stability and the durability of experimentally introduced genetic modifications across extensive passaging require further evaluation. We therefore recommend using iAMs within a defined passage range (P1–P12) with routine monitoring of key AM markers and relevant functional phenotypes, while validating critical findings in primary AMs when high biological fidelity is required. Nevertheless, iAM cells constitute a robust and versatile platform for high-throughput screening, mechanistic dissection, and genetic manipulation, which are applications that remain largely impractical with primary cells.

In summary, we report the establishment of an hTERT-immortalized mouse AM cell line that recapitulates primary-cell features more faithfully than existing models. We believe this cell line will be a valuable resource, poised to facilitate future studies on lung immunology, host defense, and pulmonary homeostasis.

## Supplementary Information

Supplementary Material 1. Supplementary Materials and Methods.

Supplementary Material 2. Fig. S1. PCR detection of mycoplasma contamination in culture supernatants of primary AMs and iAM cell lines.

Supplementary Material 3. Fig. S2.Hierarchical clustering heatmap depicting the transcriptomic relationships among the four cell types based on genome-wide expression profiling.

Supplementary Material 4. Fig. S3. Transcriptional and phenotypic characterization of iAM cells in comparison with primary alveolar macrophages. a Volcano plot showing the distribution of DEGs between iAMs and primary AMs. b KEGG pathway enrichment analysis of the combined DEG set. c Heatmap showing the expression of genes associated with AM-relevant biological pathways. d-e Flow cytometry showing the expression of F4/80 and CD11b (d) or Siglec-F and CD11c (e) in primary AM cells and iAM cells.

Supplementary Material 5. Fig. S4. iAM cells exhibit typical phagocytic activity and chemotaxis. a Flow cytometry showing the phagocytic activity of iAM cells, primary AM cells, and AM-like cells against eYFP‑labeled *E. coli*. b Cartoon showing the *in vitro* chemotaxis assay, and *P. aeruginosa* was used as a stimulus for cell migration. c Transwell cell migration assay showing the chemotaxis of iAM cells, primary AM cells, and AM-like cells *in vitro*.

Supplementary Material 6. Fig. S5. Long‑term persistence of transferred iAMs in the alveolar niche. a Flow cytometry showing CD11c^+^Siglec-F^+^ AMs in control and clodronate‑liposome‑treated mice. b Quantification of the percentage of AMs shown in (a). *n* = 6 mice per group. c Flow cytometry showing PKH26^+^CD11c^+^ and PKH26^-^CD11c^+^ cells at day 3 (left) and day 5 (right) post-transfer. Data pooled from two independent experiments are presented as mean ± SD in b. Statistical analysis was performed using a two-tailed Student’s *t*-test. Related to Fig. 3d.

Supplementary Material 7. Table S1. Comparison of STR profiles between the iAM cell line and primary AMs.

Supplementary Material 8. Table S2. Pairwise Euclidean distance matrix of transcriptomic profiles among four cell types.

## Data Availability

Bulk RNA-Seq generated for this study have been deposited in the Sequence Read Archive (SRA) under accession numbers PRJNA1471032. The data supporting the findings of this article will be shared upon reasonable request to the corresponding author.
