# Supplementary material for: hTERT-immortalized mouse alveolar macrophages retain primary-like transcriptional and functional programs
Source: Adv Biotechnol (Singap). 2026 Sep 23;4(4):37. doi: 10.1007/s44307-026-00130-x (PMC13601437; doi:10.1007/s44307-026-00130-x)
Supplement: Supplementary file 1 [file 44307_2026_130_MOESM2_ESM.pdf]

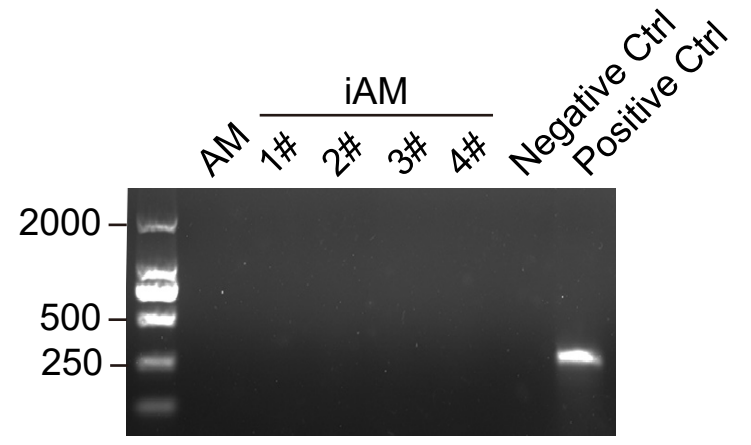
2000
500
250
AM
iAM
1#
2#
3#
4#
Negative Ctrl
Positive Ctrl
