# Supplementary material for: hTERT-immortalized mouse alveolar macrophages retain primary-like transcriptional and functional programs
Source: Adv Biotechnol (Singap). 2026 Sep 23;4(4):37. doi: 10.1007/s44307-026-00130-x (PMC13601437; doi:10.1007/s44307-026-00130-x)
Supplement: Supplementary file 2 [file 44307_2026_130_MOESM3_ESM.pdf]

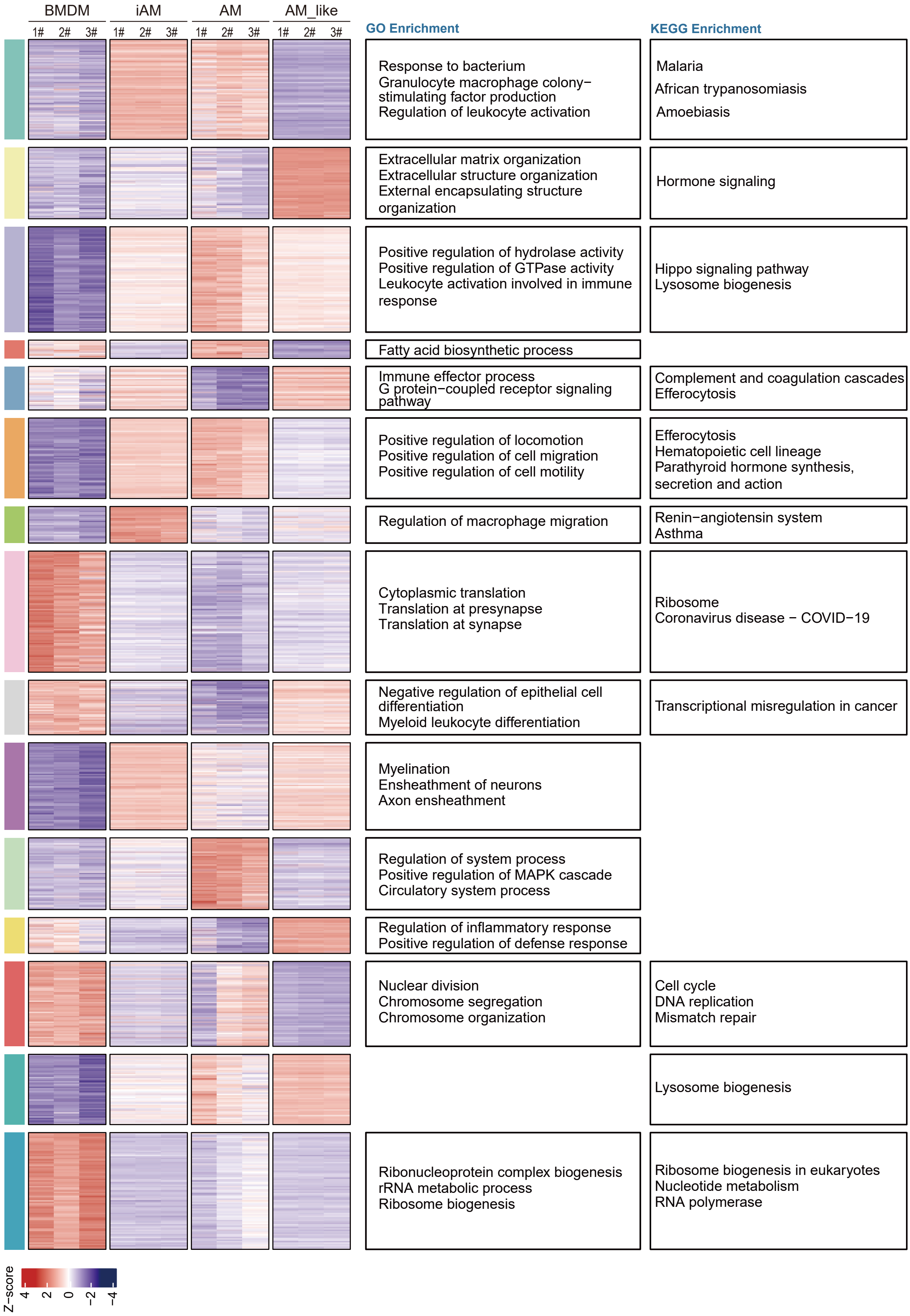
BMDM
iAM
AM
AM_like
1# 2# 3#
GO Enrichment
KEGG Enrichment
Response to bacterium
Granulocyte macrophage colony−stimulating factor production
Regulation of leukocyte activation
Malaria
African trypanosomiasis
Amoebiasis
Extracellular matrix organization
Extracellular structure organization
External encapsulating structure organization
Hormone signaling
Positive regulation of hydrolase activity
Positive regulation of GTPase activity
Leukocyte activation involved in immune response
Hippo signaling pathway
Lysosome biogenesis
Fatty acid biosynthetic process
Immune effector process
G protein−coupled receptor signaling pathway
Complement and coagulation cascades
Efferocytosis
Positive regulation of locomotion
Positive regulation of cell migration
Positive regulation of cell motility
Efferocytosis
Hematopoietic cell lineage
Parathyroid hormone synthesis, secretion and action
Regulation of macrophage migration
Renin−angiotensin system
Asthma
Cytoplasmic translation
Translation at presynapse
Translation at synapse
Ribosome
Coronavirus disease − COVID−19
Negative regulation of epithelial cell differentiation
Myeloid leukocyte differentiation
Transcriptional misregulation in cancer
Myelination
Ensheathment of neurons
Axon ensheathment
Regulation of system process
Positive regulation of MAPK cascade
Circulatory system process
Regulation of inflammatory response
Positive regulation of defense response
Nuclear division
Chromosome segregation
Chromosome organization
Cell cycle
DNA replication
Mismatch repair
Lysosome biogenesis
Ribonucleoprotein complex biogenesis
rRNA metabolic process
Ribosome biogenesis
Ribosome biogenesis in eukaryotes
Nucleotide metabolism
RNA polymerase
Z−score
4 2 0 −2 −4
