# Supplementary material for: hTERT-immortalized mouse alveolar macrophages retain primary-like transcriptional and functional programs
Source: Adv Biotechnol (Singap). 2026 Sep 23;4(4):37. doi: 10.1007/s44307-026-00130-x (PMC13601437; doi:10.1007/s44307-026-00130-x)
Supplement: Supplementary file 3 [file 44307_2026_130_MOESM4_ESM.pdf]

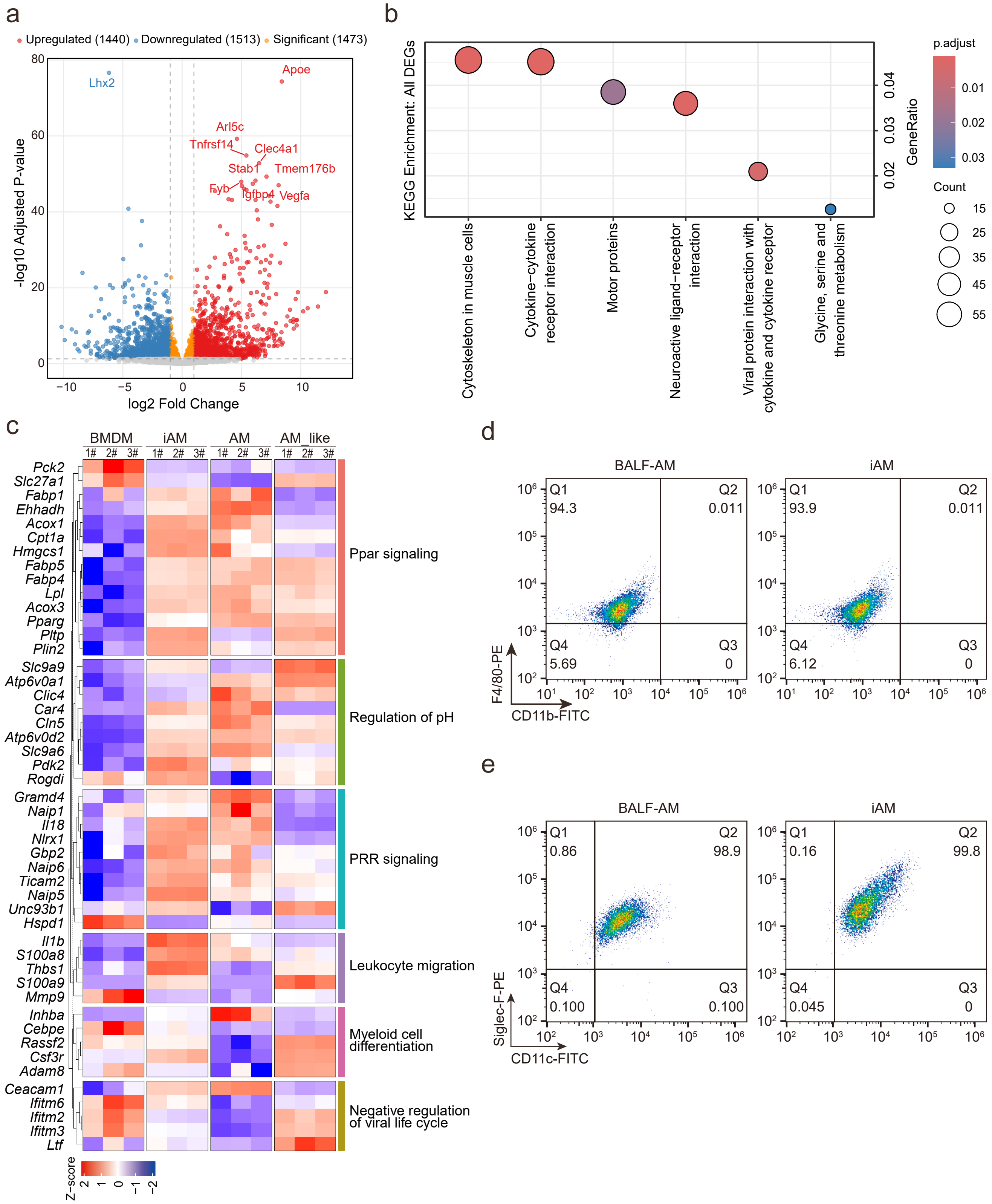
a
Upregulated (1440) Downregulated (1513) Significant (1473)
Lhx2
Apoe
Arl5c
Tnfrsf14
Clec4a1
Stab1
Tmem176b
Fyb
Igfbp4
Vegfa
-log10 Adjusted P-value
log2 Fold Change
b
KEGG Enrichment: All DEGs
Cytoskeleton in muscle cells
Cytokine−cytokine receptor interaction
Motor proteins
Neuroactive ligand−receptor interaction
Viral protein interaction with cytokine and cytokine receptor
Glycine, serine and threonine metabolism
GeneRatio
p.adjust
Count
c
BMDM
iAM
AM
AM_like
Pck2
Slc27a1
Fabp1
Ehhadh
Acox1
Cpt1a
Hmgcs1
Fabp5
Fabp4
Lpl
Acox3
Pparg
Pltp
Plin2
Ppar signaling
Slc9a9
Atp6v0a1
Clic4
Car4
Cln5
Atp6v0d2
Slc9a6
Pdk2
Rogdi
Regulation of pH
Gramd4
Naip1
Il18
Nlrx1
Gbp2
Naip6
Ticam2
Naip5
Unc93b1
Hspd1
PRR signaling
Il1b
S100a8
Thbs1
S100a9
Mmp9
Leukocyte migration
Inhba
Cebpe
Rassf2
Csf3r
Adam8
Myeloid cell differentiation
Ceacam1
Ifitm6
Ifitm2
Ifitm3
Ltf
Negative regulation of viral life cycle
Z-score
d
BALF-AM
iAM
Q1 94.3
Q2 0.011
Q4 5.69
Q3 0
Q1 93.9
Q2 0.011
Q4 6.12
Q3 0
F4/80-PE
CD11b-FITC
e
BALF-AM
iAM
Q1 0.86
Q2 98.9
Q4 0.100
Q3 0.100
Q1 0.16
Q2 99.8
Q4 0.045
Q3 0
Siglec-F-PE
CD11c-FITC
