# Supplementary material for: hTERT-immortalized mouse alveolar macrophages retain primary-like transcriptional and functional programs
Source: Adv Biotechnol (Singap). 2026 Sep 23;4(4):37. doi: 10.1007/s44307-026-00130-x (PMC13601437; doi:10.1007/s44307-026-00130-x)
Supplement: Supplementary file 4 [file 44307_2026_130_MOESM5_ESM.pdf]

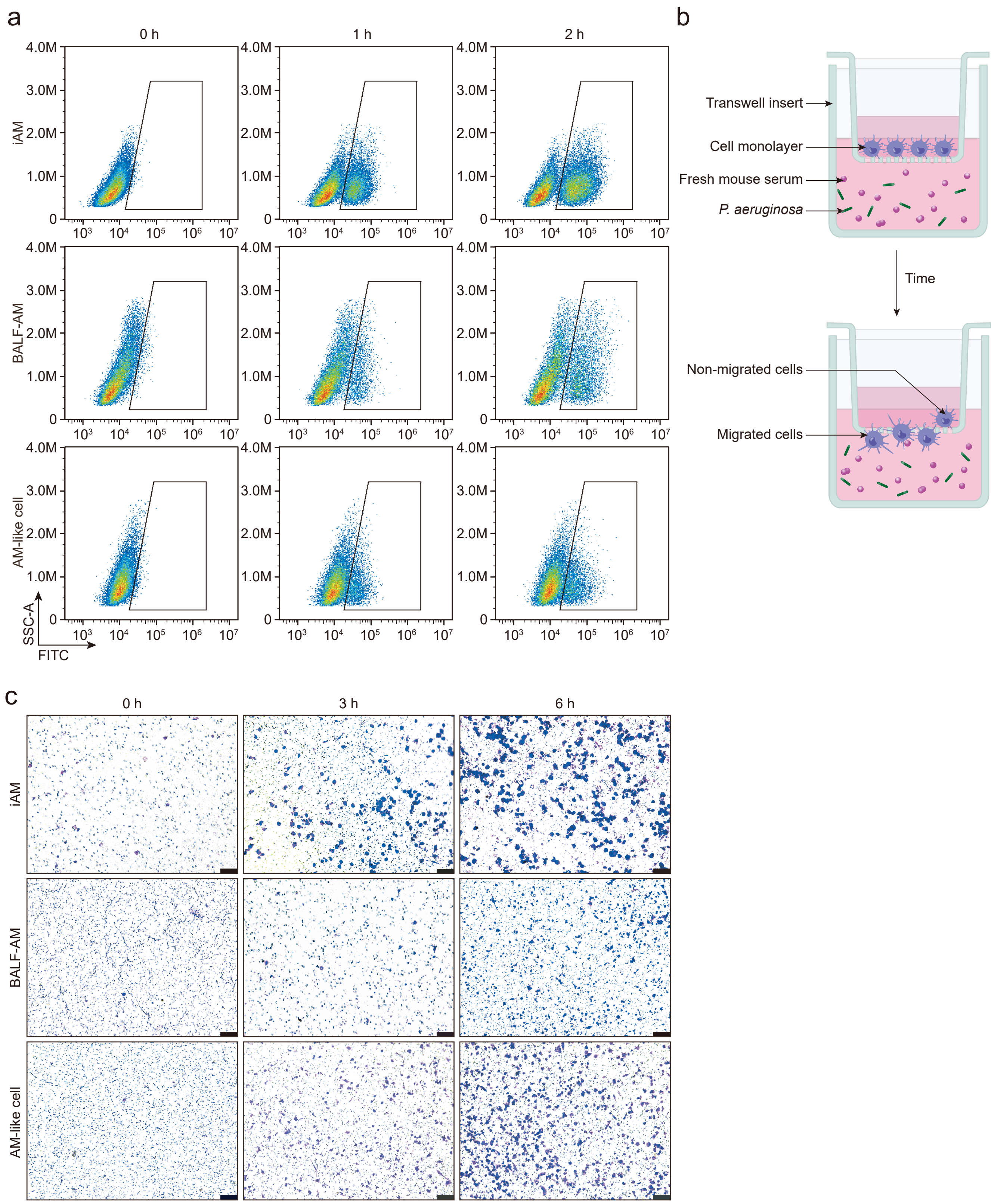
a
0 h
1 h
2 h
iAM
BALF-AM
AM-like cell
SSC-A
FITC
b
Transwell insert
Cell monolayer
Fresh mouse serum
P. aeruginosa
Time
Non-migrated cells
Migrated cells
c
0 h
3 h
6 h
iAM
BALF-AM
AM-like cell
