# Supplementary material for: hTERT-immortalized mouse alveolar macrophages retain primary-like transcriptional and functional programs
Source: Adv Biotechnol (Singap). 2026 Sep 23;4(4):37. doi: 10.1007/s44307-026-00130-x (PMC13601437; doi:10.1007/s44307-026-00130-x)
Supplement: Supplementary file 5 [file 44307_2026_130_MOESM6_ESM.pdf]

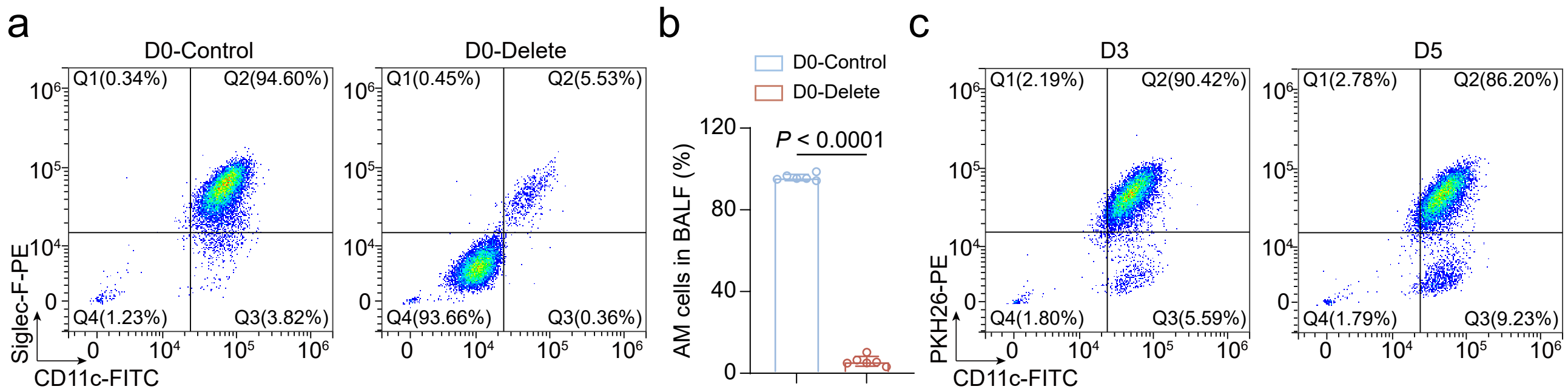
a
D0-Control
D0-Delete
Q1(0.34%)
Q2(94.60%)
Q4(1.23%)
Q3(3.82%)
Q1(0.45%)
Q2(5.53%)
Q4(93.66%)
Q3(0.36%)
Siglec-F-PE
CD11c-FITC
b
D0-Control
D0-Delete
P < 0.0001
AM cells in BALF (%)
c
D3
D5
Q1(2.19%)
Q2(90.42%)
Q4(1.80%)
Q3(5.59%)
Q1(2.78%)
Q2(86.20%)
Q4(1.79%)
Q3(9.23%)
PKH26-PE
CD11c-FITC
