# Supplementary material for: hTERT-immortalized mouse alveolar macrophages retain primary-like transcriptional and functional programs
Source: Adv Biotechnol (Singap). 2026 Sep 23;4(4):37. doi: 10.1007/s44307-026-00130-x (PMC13601437; doi:10.1007/s44307-026-00130-x)
Supplement: Supplementary file 6 [file 44307_2026_130_MOESM1_ESM.docx]

Supplemental Materials and Methods

The iAM cells used in this study were between passage 1 and passage 12 (P1–P12). For cell line authentication, STR profiling was performed at Tsingke Biotechnology Co., Ltd. Mycoplasma contamination was detected by PCR using 2× Accurate Taq Master Mix (AG11019) with universal mycoplasma primers targeting the 16S rRNA gene. For flow cytometry, cells were stained with fluorochrome-conjugated antibodies against CD11c (Cat# 11-0114-82, RRID: AB_464940), Siglec-F (Cat# 12-1702-82, RRID: AB_2637129), F4/80 (Cat# 123110, RRID: AB_893486), Ly6G (Cat# 127614, RRID: AB_2227348) and CD11b (Cat# 101207, RRID: AB_312790 or Cat# 11-0112-82, RRID: AB_464935) and analyzed on a CytoFLEX cytometer (Beckman Coulter) using FlowJo software (RRID: SCR_008520). For cytochemical staining, cells were fixed with 4% paraformaldehyde and stained with Giemsa or Oil Red O, then visualized under a light microscope. For qRT-PCR, total RNA was extracted using TRIzol (Invitrogen), and qPCR was performed on a LightCycler 480 system (Roche), with gene expression normalized to *Gapdh*. For phagocytosis, iAMs, primary AMs, and AM-like cells (Luo et al. 2021) were incubated with eYFP-expressing *E. coli* (ATCC 11303) at 10⁷ CFU for 0-2 h, and phagocytic activity was quantified by flow cytometry. For chemotaxis, cells were seeded in Transwell inserts (8-μm, Corning), with the lower chamber containing 5% fresh mouse serum and 10⁷ CFU *P. aeruginosa* (ATCC 27853). Migrated cells were stained with crystal violet and counted after 3–6 h. For RNA sequencing, libraries were sequenced on an Illumina HiSeq 2500 platform, reads were mapped to GRCm38 using HISAT2 (RRID: SCR_015530), and differentially expressed genes were identified using DESeq2 (RRID: SCR_015687) (FDR < 0.05). DEGs were defined as genes with |log_2_FC| ≥ 1 and *P*.adj < 0.05. For hierarchical clustering of samples, Euclidean distance and complete linkage method were applied using the ComplexHeatmap package (version 2.20.0, RRID: SCR_017270) in R (RRID: SCR_001905). For targeted heatmap analyses, genes were not clustered but arranged according to their module assignment. For the *in vivo* infection model, mice were challenged with 1.5 × 10^7^ CFU of *P. aeruginosa* per mouse via intratracheal instillation in 40 μL PBS. At 12 h post-infection, mice were euthanized and BALF was collected for analysis. For *in vivo* tracking, iAMs were labeled with PKH26 (Cat# PKH26PCL-1KT) prior to adoptive transfer according to the manufacturer's instructions. At days 3, 5, 7, and 14 post-transfer, BALF cells were collected and analyzed by flow cytometry to detect PKH26^+^CD11c^+^ cells. Two independent *in vivo* experiments were performed for each model, with three mice per group in each independent experiment (n = 3 per group per experiment). Mice were randomly assigned to experimental groups using a random number table. To control for potential batch effects, data from the two independent experiments were first analyzed using a two-way ANOVA with treatment and batch as between-subjects factors. No significant experiment × treatment interaction was detected, indicating that the treatment effects were consistent across the two experiments. Data from both experiments were therefore pooled for statistical analysis, resulting in a total of six independent biological replicates per group. Investigators responsible for data acquisition and quantitative analysis were blinded to group allocation where applicable. For *in vivo* experiments, representative flow cytometry plots shown in the figures are from one representative mouse from one of the two independent experiments, whereas the corresponding quantitative analyses included all mice from both experiments. For cell-based experiments, experiments were independently performed at least three times, and representative images, or flow cytometry plots shown in the figures are from one of three independent biological experiments that yielded consistent results. Statistical significance was assessed using appropriate tests (two-tailed unpaired Student’s *t*-test or one-way/two-way ANOVA with post hoc tests where applicable) as indicated in the figure legends, with a significance threshold of *P* < 0.05.
