# Supplementary material for: hTERT-immortalized mouse alveolar macrophages retain primary-like transcriptional and functional programs
Source: Adv Biotechnol (Singap). 2026 Sep 23;4(4):37. doi: 10.1007/s44307-026-00130-x (PMC13601437; doi:10.1007/s44307-026-00130-x)
Supplement: Supplementary file 7 [file 44307_2026_130_MOESM7_ESM.docx]

**Table S1.** Comparison of STR profiles between the iAM cell line and primary AMs.

| Loci | Primary AM Cells | | | iAM Cell Line | | |
| --- | --- | --- | --- | --- | --- | --- |
|  | Allele1 | Allele2 | Allele3 | Allele1 | Allele2 | Allele3 |
| STR 1-1 | 16 |  |  | 16 |  |  |
| STR 1-2 | 19 |  |  | 19 |  |  |
| STR 2-1 | 16 |  |  | 16 |  |  |
| STR 3-2 | 14 |  |  | 14 |  |  |
| STR 4-2 | 20.3 |  |  | 20.3 |  |  |
| STR 5-5 | 17 | 18 |  | 17 | 18 |  |
| STR 6-4 | 18 |  |  | 18 |  |  |
| STR 6-7 | 17 |  |  | 15 | 17 |  |
| STR 7-1 | 27.2 |  |  | 26.2 | 27.2 |  |
| STR 8-1 | 16 |  |  | 16 |  |  |
| STR 11-2 | 16 |  |  | 16 |  |  |
| STR 12-1 | 17 |  |  | 16 | 17 |  |
| STR 13-1 | 17 |  |  | 17 | 18 |  |
| STR 15-3 | 22.3 | 23.3 |  | 22.3 |  |  |
| STR 17-2 | 16 |  |  | 15 | 16 |  |
| STR 18-3 | 16 |  |  | 16 |  |  |
| STR 19-2 | 13 | 14 |  | 13 | 14 |  |
| STR X-1 | 27 |  |  | 26 | 27 |  |
| D4S2408 |  |  |  |  |  |  |
| D8S1106 |  |  |  |  |  |  |
| EV = 85.1% | | | | | | |

STR 1-1 to X-1 are mouse loci. D4S2408 and D8S1106 are human loci for contamination detection.

The Evaluation Value (EV) was calculated as: EV = (2 × number of shared alleles) / (number of alleles in query sample + number of alleles in reference sample) × 100%. According to the ATCC standards (ANSI/ATCC ASN-0002-2022), EV ≥ 80% indicates relatedness and a common ancestral origin; EV between 55% and 80% requires further verification.
