# Supplementary material for: hTERT-immortalized mouse alveolar macrophages retain primary-like transcriptional and functional programs
Source: Adv Biotechnol (Singap). 2026 Sep 23;4(4):37. doi: 10.1007/s44307-026-00130-x (PMC13601437; doi:10.1007/s44307-026-00130-x)
Supplement: Supplementary file 8 [file 44307_2026_130_MOESM8_ESM.docx]

**Table S2.** Pairwise Euclidean distance matrix of transcriptomic profiles among four cell types.

|  | AM | iAM | BMDM | AM_like |
| --- | --- | --- | --- | --- |
| AM | 20.20 | 53.89 | 99.38 | 73.89 |
| iAM | 53.89 | 1.11 | 96.22 | 60.04 |
| BMDM | 99.38 | 96.22 | 12.49 | 92.91 |
| AM_like | 73.89 | 60.04 | 92.91 | 1.96 |
